# Oral administration of *Clostridium butyricum* CGMCC0313-1 inhibits β-lactoglobulin-induced intestinal anaphylaxis in a mouse model of food allergy

**DOI:** 10.1186/s13099-017-0160-6

**Published:** 2017-02-22

**Authors:** Juan Zhang, Hui Su, Qiuhong Li, Haixia Wu, Mengyun Liu, Jianqiong Huang, Minghua Zeng, Yuejie Zheng, Xin Sun

**Affiliations:** 1 Department of Pediatrics, Xijing Hospital, the Fourth Military Medical University, Xi’an, 710032 China; 2 Department of Geratology, Xijing Hospital, the Fourth Military Medical University, Xi’an, 710032 China; 3 Respiratory Department, Shenzhen Children’s Hospital, Shenzhen, 518036 China

**Keywords:** β-Lactoglobulin, *Clostridium butyricum*, Food allergy, Mice, Probiotics

## Abstract

**Background:**

Probiotic bacteria can induce immune regulation or immune tolerance in patients with allergic diseases, but the underlying mechanisms are still unclear. There has been a growing interest in the use of beneficial bacteria for allergic diseases recently. This study aimed at exploring whether *Clostridium butyricum* CGMCC0313-1 (*C. butyricum*) can reduce β-lactoglobulin(BLG)-induced intestinal anaphylaxis in a murine model of food allergy.

**Methods:**

The preventive and therapeutic effects of oral *C. butyricum* on anaphylactic symptoms induced via BLG in food allergy mice were investigated. Intestinal anaphylaxis, T helper (Th)-specific cytokines and transcription factors, secretory IgA (sIgA), CD4+ CD25+ Foxp3Treg cell and histopathological alterations were examined.

**Results:**

*Clostridium butyricum* significantly ameliorated intestinal anaphylaxis symptoms in the food allergy mice. sIgA and CD4+ CD25+ Foxp3Treg cell were increased by oral *C. butyricum*. It also reversed the imbalance of Th1/Th2 andTh17/Treg.

**Conclusions:**

*Clostridium butyricum* reduces BLG-induced intestinal anaphylaxis in mice and might be an additional or supplementary therapy for food allergy.

## Background

Food is a foreign antigen that is necessary for nutrition. Inevitably, food antigens present a continuous challenge throughout life. Humans have adapted to food via mechanisms of immune tolerance. Food allergy is defined as abnormal immune responses resulting from breakdown of natural oral tolerance [1]. Food allergy is an increasing public health problem worldwide [2–5] and has been estimated to affect approximately 5% of adults and 8% of children [6]. Among food allergies, cow’s milk allergy is one of the earliest and most prevalent food allergies and β-lactoglobulin (BLG) is the major allergen [7, 8]. Diets based on cow’s milk play a major role in children’s nutrition as it has an essential effect on the patient’s quality of life. Additionally, up to now the current standard for prevention of food allergy is still the strict allergen avoidance and the elimination of the triggering food from the diets [9]. However, accidental ingestion is difficult to be absolutely avoided in our life. Therefore, effectively prevent and manage food allergy to restore immune tolerance is particularly needed.

A previous study shows that the increased prevalence of allergic diseases is associated with decreased microbial exposure and alteration of microbial communities represented in the gut microbiota [10]. It has been demonstrated that the composition and metabolic activity of the microbiota is crucial for both the maintenance and the development of immune homeostasis, as well as the induction of immune tolerance [11, 12]. Intervention strategies targeting the intestinal microbiota include the deliberate administration of probiotic bacteria. Probiotics are live microorganisms that confer a health benefit to the host when administered in adequate amounts [13]. Different bacterial strains or their mixtures have been used to assess their protective effects for allergic diseases in clinical trials, but the results have been controversial [14–16]. Possible mechanisms of their protective action include both the induction of regulatory dendritic cells (regDCs) and T cells and the skewing the imbalances of T helper (Th)1/Th2 as well as Th17/Treg, together with the enhancement of the epithelial barrier function [10, 17–21]. Imbalances in Th responses can also be detected using Th-specific transcription factors: T-bet for Th1 cells, GATA-3 for Th2 cells, retinoic acid orphan receptor-γt (RORγt) for Th17 cells and forkhead box P3 (Foxp3) for Tregs [22]. Nevertheless, the knowledge of molecular mechanisms underlying probiotic-host interactions that shape host immune system in a protective setting is still incomplete. Mouse models of food allergy to clinically relevant allergens could be helpful in providing information difficult to be obtained in human studies.

*Clostridium butyricum* CGMCC0313-1 (*C. butyricum*) has been widely used for improving gastrointestinal function as probiotics [23, 24]. However, the benefit of *C. butyricum* on food allergy is rarely reported. This study aimed to explore whether oral *C. butyricum* can reduce food allergy in mice. Findings from this study will contribute to a better understanding of the protective effects of the beneficial bacteria in allergic diseases.

## Methods

### Mice

Male BALB/c mice of 6–8 weeks were purchased from the Laboratory Animal Center of the Fourth Military Medical University and acclimated to their new environment for 1 week. Animals were housed under conventional conditions, fed standard mouse pellets and water *adlibitum*. Experimental procedures were approved by the Ethics Committee for Animal Studies of the Fourth Military Medical University (20150901) and performed in accordance with their guidelines of the Institutional Animal Care and Use Committee.

### The probiotics

The *C. butyricum* powder (Kexing Biotech Company limited, Weifang, Shandong, China) was stored at −20 °C. Drinks were prepared using normal saline (NS) only or NS plus *C. butyricum*. The concentration of *C. butyricum* was 5 × 10^8^ CFU/ml.

### Mouse model of food allergy

**Fig. 1:**
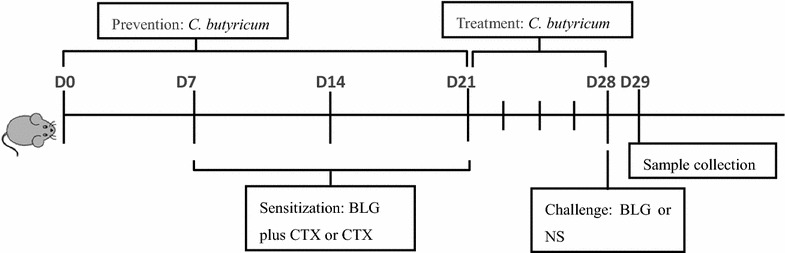
The protocols used for the mouse models of food allergy. Male BALB/c mice were sensitized orally with BLG plus CTX or only CTX on day 7, 14 and 21, and followed by oral administration of 100 mg BLG challenge on day 28. The animals received *C. butyricum* feeding from day 1 to day 21 or from day 22 to day 28 by oral gavage

BALB/c mice were randomly assigned into 4 experimental groups with 10 in each group. The 4 groups of mice were treated as follow: the food allergy group received 20 mg BLG (Sigma, St. Louis, MO, USA) plus 10 μg cholera toxin(CTX, Sigma) orally on days 7, 14 and 21, and followed by oral administration of 100 mg BLG challenge on day 28; the preventive and treated groups were approached as the food allergy group, and the animals were given 200 μl *C. butyricum* feeding from days 1 to 21 or from days 22 to 28 respectively; mice from the control group only received 10 μg CTX orally, and followed by challenge with NS (Fig. 1).

### Measurement of intestinal anaphylaxis

Intestinal anaphylaxis was assessed in challenged mice by the allergic diarrhea and circulating mouse mast cell protease 1 (mMCP-1) levels as previously described [25, 26]. Briefly, mice were observed for the presence of diarrhea for 1 h after the challenge and were scored as diarrhea positive or negative. Diarrhea and anaphylactic symptoms were scored by visually monitoring mice for 60 min after challenge. Diarrhea—0, normal stools; 1, a few wet and unformed stools; 2, a number of wet and unformed stools with moderate perianal staining of the coat; 3, severe, watery stool with severe perianal staining of the coat. Anaphylactic symptoms—0, no symptoms; 1, reduced activity, trembling of limbs; 2, loss of consciousness, no activity upon prodding; 3, convulsions, death. Serum was obtained 1 h following the final antigen challenge for measurement of mMCP-1 using the commercial enzyme-linked immunosorbentassay (ELISA) (eBioscience, San Diego, CA, USA) according to manufacturer instructions (Table 1).

**Table 1:** Scoring methods of diarrhea and anaphylactic symptoms

Diarrhea	Anaphylactic symptoms	Scores
Normal stools	No symptoms	0
A few wet and unformed stools	Reduced activity, trembling of limbs	1
A number of wet and unformed stools with moderate perianal staining of the coat	Loss of consciousness, no activityupon prodding	2
Severe, watery stool with severe perianal staining of the coat	Convulsions, death	3

### The measurement of cytokines and immunoglobulin

Serum samples were collected to assay the presence of cytokines and immunoglobulin (Ig). Levels of serum IL-4, IL-5, IL-13, IL-17, INF-γ, IL-10, TGF-β1 and the total IgE were measured by ELISA Kits (RD system, Boston, MA, USA; Uscn Life, Wuhan, Hubei, China) following the manufacture’s protocol.

### Secretory IgA

Small intestine was rinsed with 10 ml of cold PBS. Intestinal lavages were centrifuged at 12,000×*g* for 20 min at 4 °C, and levels of secretory IgA (sIgA) in the supernatants were determined by ELISA (Uscn Life) as previously described [27].

### RNA isolation and quantitative real-time PCR

After mice were sacrificed on day 29, the spleen was dissected and immersed in RNAlater® (Ambion, Austin, TX, USA) for PCR. Total RNA (n = 6 mice per group) was carried out using Trizol (Ambion) from whole spleen tissue and reverse-transcribed to cDNA by a PrimeScript™ RT Master Mix Kit (TaKaRa, Tokyo, Japan). cDNA was amplified using SYBR® Premix *Ex Taq*™ II Kit (TaKaRa) and run in the Real-Time PCR Detection System. Primers for T cell transcription factors were designed and synthesized by TaKaRa Biotechnology (Dalian, Liaoning, China). The sequences were listed in Table 2. The relative levels of gene expression were calculated by reference to the level of β-actin using the ΔΔCT method **[** 28 **]**.

**Table 2:** Oligonucleotide primers used in the study

T-bet(F)	5′-CATGGAGAACGGAGAATGGA-3′
T-bet(R)	5′-TGGACAGGGGAAGAGAGCA-3′
RORγt(F)	5′-GCTCCATATTTGACTTTTCCCACT-3′
RORγt(R)	5′-GATGTTCCACTCTCCTCTTCTCTTG-3′
GATA-3(F)	5′-GGATTTAAGTCGAGGCCCAAG-3′
GATA-3(R)	5′-ATTGCAAAGGTAGTGCCCGGTA-3′
Foxp3(F)	5′-CCCAGGAAAGACAGCAACCTT-3′
Foxp3(R)	5′-TTCTCACAACCAGGCCACTTG-3′
β-actin(F)	5′-CATCCGTAAAGACCTCTATGCCAAC-3′
β-actin(R)	5′-ATGGAGCCACCGATCCACA -3′

*F* forward, *R* reverse

### Flow cytometry

Single-cell suspensions isolated from mesenteric lymph nodes (MLN) were stained for FACS analyses as described previously [29]. Cells were first stained for surface markers including CD4-PerCP-Cy5.5, CD25-FITC (BD Pharmingen, San Diego, CA, USA). If required, cells were then fixed and permeabilized by BD Cytofix/Cytoperm reagent (BD Bioscience, San Jose, CA, USA) and stained for intracellular expression markers, Foxp3-PE. Data were acquired with FACSCanto (Beckman Coulter, Miami, FL, USA) and analyzed by FlowJo 10.0.7 software.

### Histological analysis

Paraffin-embedded sections of proximal jejunum were stained with hematoxylin and eosin (H&E) for morphological analysis. Villus length was determined by measuring the distance in μm from the crypt neck to the villus tip using Image J software. Six animals from each experimental group were evaluated, and a minimum of 12 well-oriented villi from each section were measured. Data were reported as villus size measured in μm. Morphological analyses were performed in a blinded manner to prevent observer bias.

### Statistical analysis

Data were expressed as the mean ± standard error means (SEMs). All data were analyzed with SPSS17.0 software (SPSS Inc, Illinois, Chicago, USA). One-Way ANOVA and Mann–Whitney *U* non-parametric test was conducted to determine the statistical significance, where appropriate. A *P* value of <0.05 was considered statistically significant.

## Results

### *Clostridium butyricum* ingestion inhibits the development of intestinal anaphylaxis

**Fig. 2:**
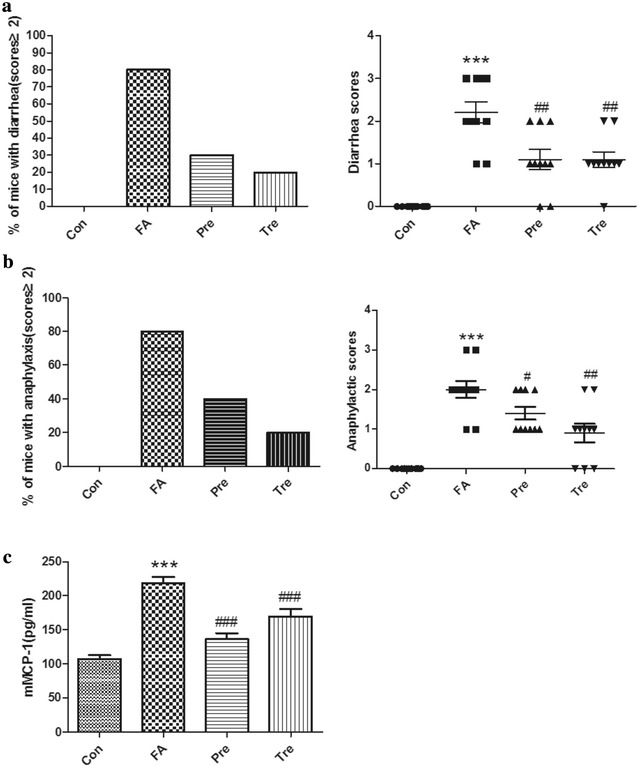
*Clostridium butyricum* inhibits the development of intestinal anaphylaxis. Diarrhea occurrence, diarrhea scores (**a**), anaphylactic response, anaphylactic score (**b**) and levels of mMCP-1 (**c**) were monitored. Male BALB/c mice were given NS or sensitized/challenged with BLG ± treatment with *C. butyricum*. Con, n = 10 for the control group; FA, n = 10 for the food allergy group; Pre, n = 10 for the preventive group; and Tre, n = 10 for the treated group. Results are shown as means ± SEMs. *P < 0.05, **P < 0.01, ***P < *0.001* versus the control group, and ^*#*^ *P* < *0.05,* ^*##*^ *P* < *0.01,* ^*###*^ *P* < *0.001* versus the food allergy group

Oral challenges with BLG in sensitized mice lead to increasing severity of diarrhea and anaphylaxis, and a robust increase in circulating mMCP-1. Administration of *C. butyricum* obviously inhibited BLG-induced food allergy, namely diarrhea occurrence and scores (Fig. 2a), anaphylactic response and scores (Fig. 2b), and levels of mMCP-1 (Fig. 2c), suggesting a protective effect of *C. butyricum* on food allergy in mice.

### Cytokines and immunoglobulin

**Fig. 3:**
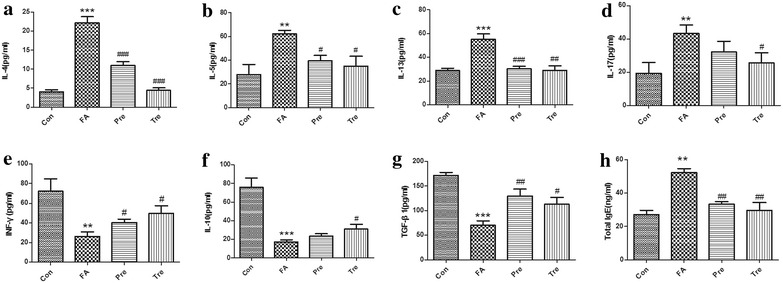
The effect of *C. butyricum* on cytokines and immunoglobulin. Levels of serum IL-4 (**a**), IL-5 (**b**), IL-13 (**c**), IL-17 (**d**), INF-γ (**e**), IL-10 (**f**), TGF-β1 (**g**) and the total IgE (**h**) were measured by ELISA Kits. Male BALB/c mice were given NS or sensitized/challenged with BLG ± treatment with *C. butyricum*. Con, n = 10 for the control group; FA, n = 10 for the food allergy group; Pre, n = 10 for the preventive group; and Tre, n = 10 for the treated group. Results are shown as means ± SEMs. *P < 0.05, **P < 0.01, ***P < *0.001* versus the control group, and ^*#*^ *P* < *0.05,* ^*##*^ *P* < *0.01,* ^*###*^ *P* < *0.001* versus the food allergy group

Cytokines and total IgE were determined using ELISA Kits (Fig. 3). Compared with the control group, the levels of inflammatory cytokines in the serum (Fig. 3a–d: IL-4, IL-5, IL-13, IL-17) were significantly increased in the food allergy group and decreased in the preventive and treated groups. However, the IL-17 of the preventive group was not significant different from that of the food allergy group (*P* > 0.05). Levels of INF-γ, IL-10 and TGF-β1 (Fig. 3e–g) in the food allergy group were decreased compared with the counterparts of the control group. They increased in the groups received *C. butyricum* orally except for the IL-10 in the preventive group.

Compared with the control group, the total IgE (Fig. 3h) was significantly elevated in the food allergy group (*P* < 0.01). However, they decreased dramatically in the preventive and treated group.

### *Clostridium butyricum* supress the intestinal levels of sIgA

**Fig. 4:**
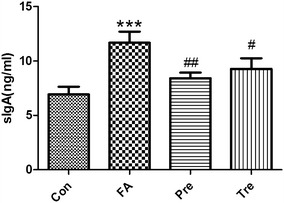
*Clostridium butyricum* supress the intestinal levels of sIgA. Levels of sIgA were determined by ELISA. Male BALB/c mice were given NS or sensitized/challenged with BLG ± treatment with *C. butyricum*. Con, n = 10 for the control group; FA, n = 10 for the food allergy group; Pre, n = 10 for the preventive group; and Tre, n = 10 for the treated group. Results are shown as means ± SEMs. *P < 0.05, **P < 0.01, ***P < *0.001* versus the control group, and ^*#*^ *P* < *0.05,* ^*##*^ *P* < *0.01,* ^*###*^ *P* < *0.001* versus the food allergy group

Since sIgA contributes to the barrier function and it is involved in immunological homeostasis [30], we measured levels of this immunoglobulin in small intestinal lavage fluids. sIgA levels were augmented in allergic mice following local inflammation. *C. butyricum* was effective in suppressing such augment in the preventive and treated group (Fig. 4).

### *Clostridium butyricum* results in a strong up-regulation of mRNA for Tbet and Foxp3

**Fig. 5:**
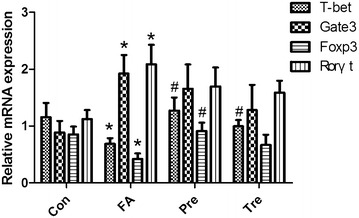
*Clostridium butyricum* up-regulates Tbet and Foxp3 mRNA expression. The mRNA expression of Th specific transcription factors was measured by quantitative real-time PCR. Male BALB/c mice were given NS or sensitized/challenged with BLG ± treatment with *C. butyricum*. Con, n = 10 for the control group; FA, n = 10 for the food allergy group; Pre, n = 10 for the preventive group; and Tre, n = 10 for the treated group. Results are shown as means ± SEMs. *P < 0.05, **P < 0.01, ***P < *0.001* versus the control group, and ^*#*^ *P* < *0.05,* ^*##*^ *P* < *0.01,* ^*###*^ *P* < *0.001* versus the food allergy group

To further explore the effects of the different interventions on Th responses in the spleen, the mRNA expression of Th specific transcription factors was measured (Fig. 5). The expression of Th1-(Tbet) and Treg-(Foxp3) transcription factors was significantly decreased in the food allergy group as compared to the control group, whereas the expression of Th2-(Gata3) and Th17-(Rorγt) transcription factors increased dramatically. However, Tbet and Foxp3 expression were significantly increased in the preventive group. Compared to the food allergy group, Tbet expression was increased in the treated group, however, the expression of Foxp3 remained unchanged.

### *Clostridium butyricum* skews the immune response away from Th2 and towards Treg

**Fig. 6:**
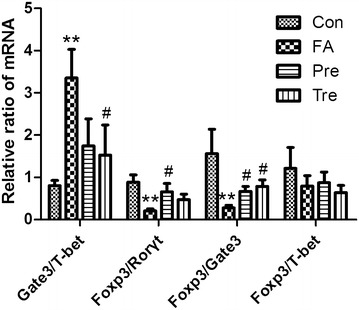
*Clostridium butyricum* skews the immune response away fromTh2 and towards Treg. Ratios for Gata3/Tbet (Th2/Th1), Foxp3/Rorγt (Treg/Th17), Foxp3/Gata3 (Treg/Th2) and Foxp3/Tbet (Treg/Th1) mRNA expression in whole spleen tissue are shown for each *C. butyricum* treatment. Male BALB/c mice were given NS or sensitized/challenged with BLG ± treatment with *C. butyricum*. Con, n = 10 for the control group; FA, n = 10 for the food allergy group; Pre, n = 10 for the preventive group; and Tre, n = 10 for the treated group. Results are shown as means ± SEMs. *P < 0.05, **P < 0.01, ***P < *0.001* versus the control group, and ^*#*^ *P* < *0.05,* ^*##*^ *P* < *0.01,* ^*###*^ *P* < *0.001* versus the food allergy group

To determine the extent of Th response skewing in the spleen, ratios for Gata3/Tbet (Th2/Th1), Foxp3/Rorγt (Treg/Th17), Foxp3/Gata3 (Treg/Th2) and Foxp3/Tbet (Treg/Th1) mRNA expression were calculated (Fig. 6). The food allergy groups showed a Th2-skewed immune response represented by a significant increase in Gata3/Tbet ratio as compared to control group. *C. butyricum* reduced this ratio in the treated group, but not the preventive group. Interestingly, the Foxp3/Rorγt (except for the treated group)and Foxp3/Gata3 ratios were significantly increased in the groups received *C. butyricum* orally as compared to the food allergy group indicating an increase in Treg-associated responses. The ratio of Foxp3/Tbet did not differ significantly among the different treatment groups.

### The effect of *C. butyricum* on Treg cell

**Fig. 7:**
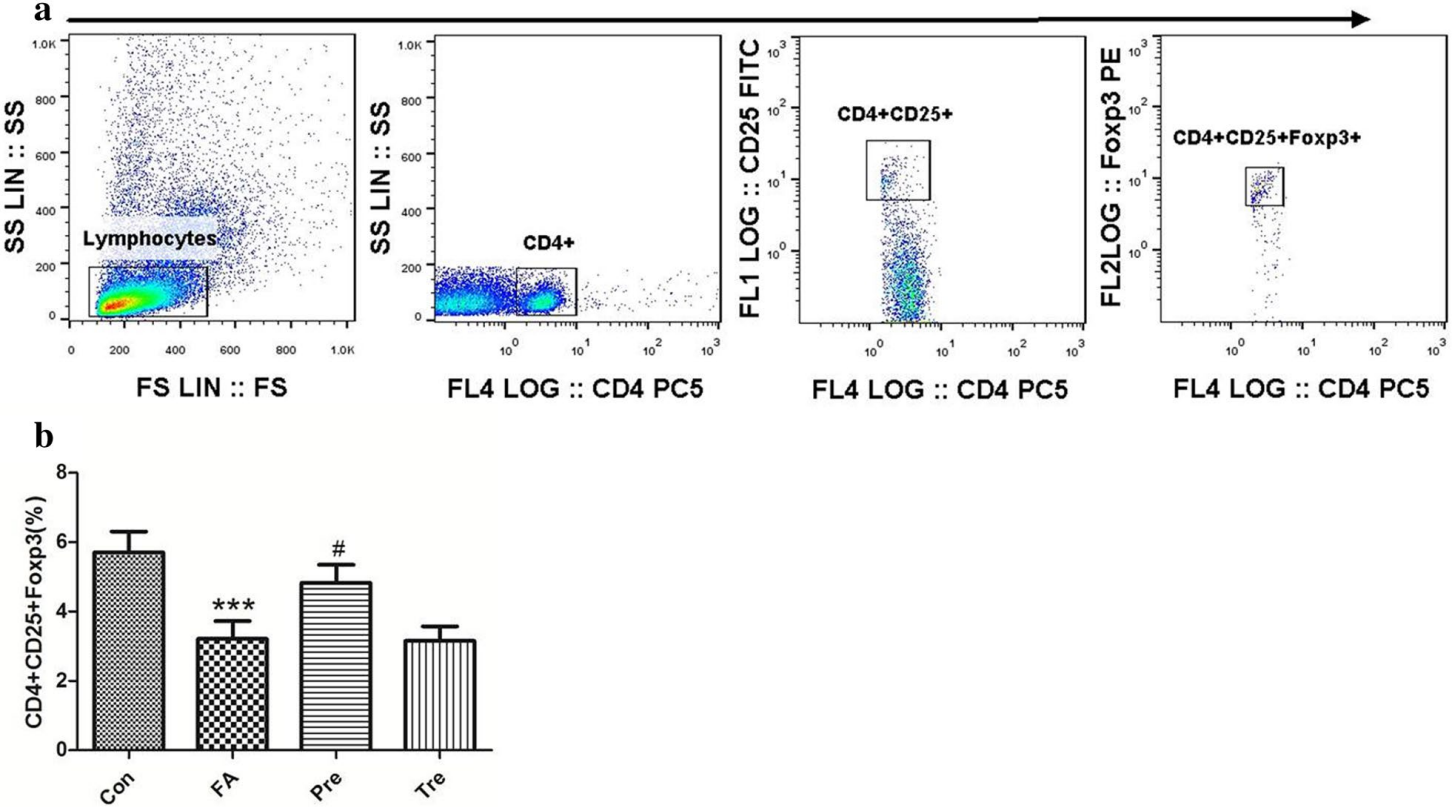
The effect of *C. butyricum* on Treg cell. The gating strategy (**a**) and effect of oral administration of *C. butyricum* on populations of CD4+ CD25+ Foxp3+ (**b**) cells in the MLN. Male BALB/c mice were given NS or sensitized/challenged with BLG ± treatment with *C. butyricum*. Con, n = 10 for the control group; FA, n = 10 for the food allergy group; Pre, n = 10 for the preventive group; and Tre, n = 10 for the treated group. Results are shown as means ± SEMs. *P < 0.05, **P < 0.01, ***P < *0.001* versus the control group, and ^*#*^ *P* < *0.05,* ^*##*^ *P* < *0.01,* ^*###*^ *P* < *0.001* versus the food allergy group

We examined the potential induction of Foxp3 by regulatory T cells in the MLN of the mice. Food allergy mice had a lower percentage of CD4+ CD25+ Foxp3+ T cells in the MLN compared to nonsensitized controls. The numbers of CD4+ CD25+ Foxp3+ Treg cells was increased in the preventive group, however, no significant differences in these regulatory T-cell populations were observed in the treated group as compared to the food allergy group (Fig. 7).

### *Clostridium butyricum* reduces histological changes in allergic mice

**Fig. 8:**
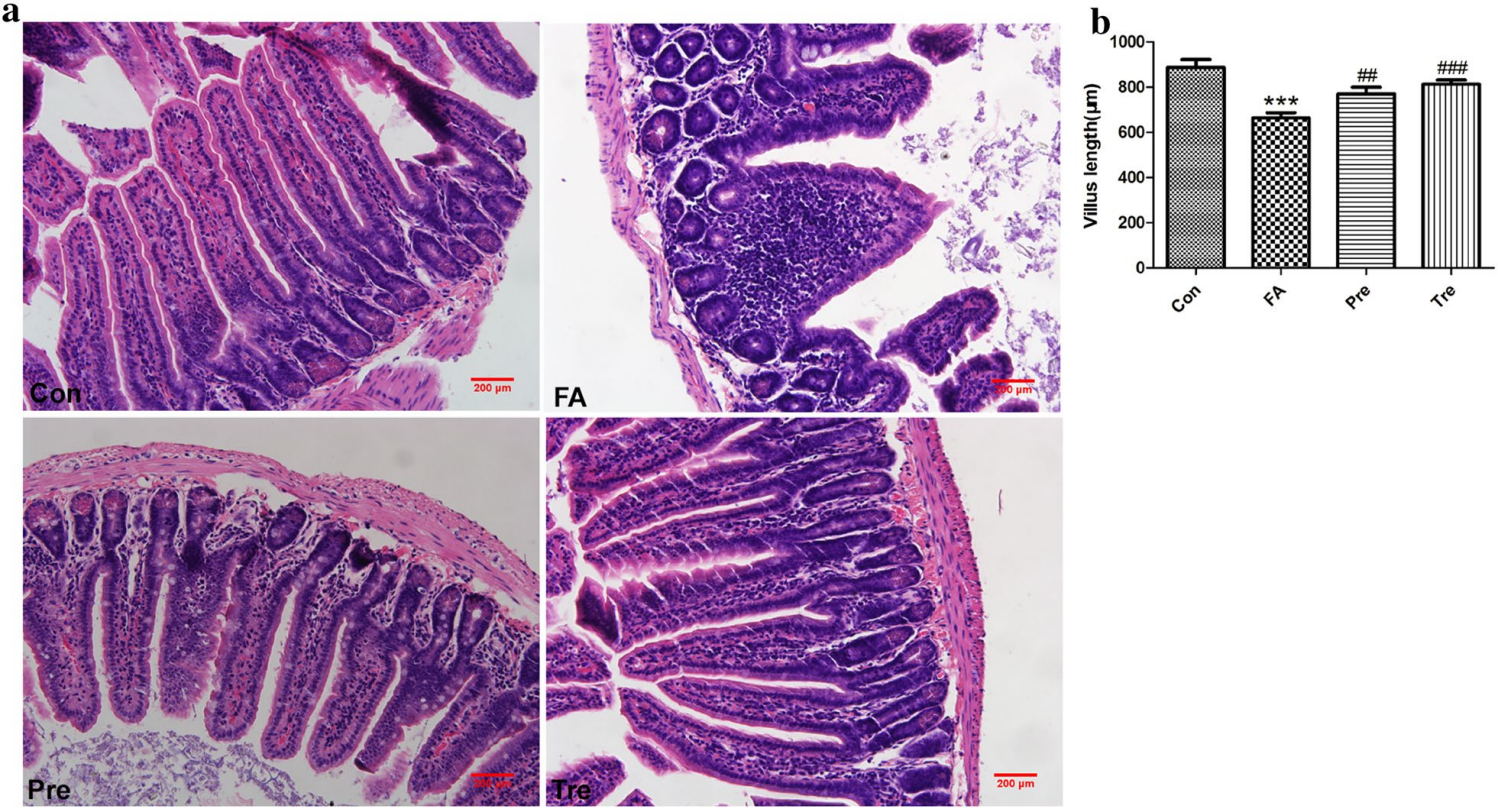
*Clostridium butyricum* reduces histological changes in allergic mice. Representative H&E staining of intestinal sections (**a**, 200× magnification).* Bar graph* of villus height (**b**) determined by measuring vertically well-oriented crypt villus units from H&E-stainedsections of mice. Male BALB/c mice were given NS or sensitized/challenged with BLG ± treatment with *C. butyricum*. Con, n = 10 for the control group; FA, n = 10 for the food allergy group; Pre, n = 10 for the preventive group; and Tre, n = 10 for the treated group. Results are shown as means ± SEMs. *P < 0.05, **P < 0.01, ***P < *0.001* versus the control group, and ^*#*^ *P* < *0.05*, ^*##*^ *P* < *0.01*, ^*###*^ *P* < *0.001* versus the food allergy group

Ingestion of BLG induced submucosal edema and increased inflammatory cell infiltration in the gut of sensitized mice (Fig. 8a). Allergic mice had decrease villus height whereas *C. butyricum*-treatment animals had a similar alteration to the one found in the control mice (Fig. 8b). Thus, oral administration of *C. butyricum* reduced the development of intestinal inflammation.

## Discussion

Food allergy is an increasing health problem and it has a significant impact on the health and daily activities of allergic individuals. The aim of this study was to investigate the preventive and therapeutic effect of *C. butyricum* on food allergy in mice. In our study, there were higher scores about diarrhea and anaphylactic symptoms in the food allergy group as compared to the control group. Positive incidence of mice with allergic diarrhea and anaphylactic symptoms was increased in the food allergy mice. Additionally, the expression of mMCP-1 and total IgE were significantly increased in the allergic mice. These were similar to the results observed in a study of ovalbumin allergy [31], however, we lack the data about BLG specific IgE due to the unavailable of the kit from the manufacturer. More importantly, decreased villus length and increased inflammatory reaction were observed in the gut slides of the food allergy mice. Hence, we successfully mimic BLG-induced allergic intestine inflammation in our mouse model.

Various strains of probiotics have been used in animal models of food allergy. Probiotic VSL#3-induced TGF-β ameliorates food allergy inflammation in a mouse model of peanut sensitization through the induction of regulatory T cells in the gut mucosa [32]. A combination of specific immunotherapy with *C. butyricum* significantly enforces the therapeutic effect on inhibiting the food allergen related inflammation in the intestine, which can be a novel approach for the treatment of food allergy [33]. A recent study suggests that oral supplementation of *Lactobacillus paracasei* L9 (L9) can reduce the development of allergic sensitization to BLG, likely through regDCs mediated active suppression [34]. In a BALB/c mouse model of BLG allergy, oral administration of *Lactobacillus acidophilus* (*L. acidophilus*)can suppress the major allergic symptoms probably due to improve the regulatory T (Treg)/Th17 balance and inhibit the IL-6 production [35]. It has been confirmed that *Bifidobacterium longum* BBMN68 (BBMN68) may be a suitable therapeutic approach to the alleviation of food allergies likely through the specific induction of CD11c+ CD103+ DCs and semi-mature DCs [36]. Similarly, oral administration of *C. butyricum* to the mouse models of food allergy is effective in reducing allergic inflammation in our study.

Th2 cells play a key role in the pathogenesis of food allergy, and patients with food allergy were reported to have Th1/Th2 imbalances as well as disturbed Th17/Treg balances. Th2 cytokines including IL-4, IL-5, IL-13 [37]. IFN-γ released by Th1 can inhibit the development of Th2. Th17 cells release IL-17 which is connected with inflammation in the gut [38, 39]. High levels of IL-10, TGF-β expressed by regDCs can directly mediate the conversion of T cells into Foxp3+ Treg cells and induce immune tolerance [17]. Th2 dominance was observed in the food allergy group represented by a significant decrease in Th1 and Treg transcription factors and high Gata3/Tbet ratio. Hence, our model mimics the Th2-responses found in food allergy. Importantly, *C. butyricum* shifted the immune balance towards Th1 and Treg, with significantly increased Foxp3/Rorγt and Foxp3/Gata ratios and a significantly decreased Gata3/Tbet ratio. These findings are consistent with results of those previous studies. BBMN68 and L9 has been already shown to significantly reduce BLG-specific hypersensitivity reactions by suppressing the aberrant balance of Th1/Th2 responses with increasing the number of CD4+ CD25+ Foxp3+ Treg cells [34, 40]. *L. acidophilus* supplementation is capable of reducing allergic symptoms in a mouse model of food allergy through reversing the imbalance of regulatory T (Treg)/Th17. More importantly, the above observed effects of beneficial bacteria on the Th responses are mirrored by the detection of CD4+ CD25+ Foxp3+ Tregs in the MLN of the animals. Tregs play a key role in balancing immune responses and it was demonstrated that increased expression of Foxp3 in Tregs is directly associated with increased function of these cells [41]. Preventive ingestion of *C. butyricum* increase the populations of CD4+ CD25+ Foxp3+ cells in the MLN and induced high levels of TGF-β and IL-10 in the serum, indicating the powerful effect of probiotics on modulating the intestinal immune response. These were similar to results observed in a study of ovalbumin allergy, which showed that LGG only induced the number of CD4+ CD25+ Foxp3+ Treg cells and TGF-β secretion [42]. However, No significant differences in the percentage of CD4+ CD25+ Foxp3+ cells were observed in the treated group as compared with the food allergy group.

sIgA plays a protective role by antigen binding and exclusion [43]. Food allergy may cause impaired epithelial barrier function, including sIgA release into the gut lumen [44]. We observed enhanced levels of sIgA in feces of the food allergy mice. sIgA can mediate a potent anti-inflammatory function following the interaction with SIGNR1 on DC which induces an immune tolerance via regulatory T cell expansion [45]. Therefore, the increase of the protective secretory immunoglobulin might be a regulatory mechanism triggered to counteract allergic inflammation to BLG in the gut mucosa. Levels of sIgA enhanced in allergic mice suggesting that inflammation triggered this modulatory component of immune response. The groups received *C. butyricum* orally, on the other hand, had little inflammatory consequences and therefore were not accompanied by augmented levels of sIgA.

## Conclusions

To our knowledge, this is the first report in which the preventive and therapeutic effects of *C. butyricum* on food allergy were investigated. The findings reported here indicate that oral probiotics such as *C. butyricum*, with established anti-inflammatory and anti-allergic activity, can significantly modulate the mucosal immune response and may represent an effective and safe strategy for patients with food allergies, for which no established and effective cures based on the pathogenetic mechanisms are available.

## Abbreviations

*C. butyricum*: *Clostridium butyricum* CGMCC0313-1
BLG: ß-lactoglobulin
Th: T helper
sIgA: secretory IgA
DC: dendritic cells
RORγt: retinoic acid orphan receptor-γt
Foxp3: forkhead box P3
CTX: cholera toxin
NS: normal saline
mMCP-1: mouse mast cell protease 1
ELISA: enzyme-linked immunosorbent assay
Ig: immunoglobulin
MLN: mesenteric lymph nodes
H&E: hematoxylin and eosin
L9: *Lactobacillus paracasei* L9
regDCs: regulatory dendritic cells
*L. acidophilus*: *Lactobacillus acidophilus*
BBMN68: *bifidobacterium longum* BBMN68
SEMs: standard error means
